# Hypes and Hopes of Stem Cell Therapies in Dentistry: a Review

**DOI:** 10.1007/s12015-021-10326-4

**Published:** 2022-01-11

**Authors:** Alessandra Rodriguez y Baena, Andrea Casasco, Manuela Monti

**Affiliations:** 1 Program in Biomedical Sciences and Engineering, Department of Molecular, Cell, and Developmental Biology, University of California-Santa Cruz, Santa Cruz, CA 95064 USA; 2 Department of Public Health, Experimental and Forensic Medicine, Histology and Embryology Unit, University of Pavia, Pavia, Italy; 3 Dental & Face Center, CDI, Milan, Italy; 4 Research Center for Regenerative Medicine, Fondazione IRCCS Policlinico San Matteo, Pavia, Italy

**Keywords:** Dental stem cells, Spheroids, Organoids, Biomaterials, Regenerative medicine

## Abstract

One of the most exciting advances in life science research is the development of 3D cell culture systems to obtain complex structures called organoids and spheroids. These 3D cultures closely mimic in vivo conditions, where cells can grow and interact with their surroundings. This allows us to better study the spatio-temporal dynamics of organogenesis and organ function. Furthermore, physiologically relevant organoids cultures can be used for basic research, medical research, and drug discovery. Although most of the research thus far focuses on the development of heart, liver, kidney, and brain organoids, to name a few, most recently, these structures were obtained using dental stem cells to study in vitro tooth regeneration. This review aims to present the most up-to-date research showing how dental stem cells can be grown on specific biomaterials to induce their differentiation in 3D. The possibility of combining engineering and biology principles to replicate and/or increase tissue function has been an emerging and exciting field in medicine. The use of this methodology in dentistry has already yielded many interesting results paving the way for the improvement of dental care and successful therapies.

**Graphical abstract:**

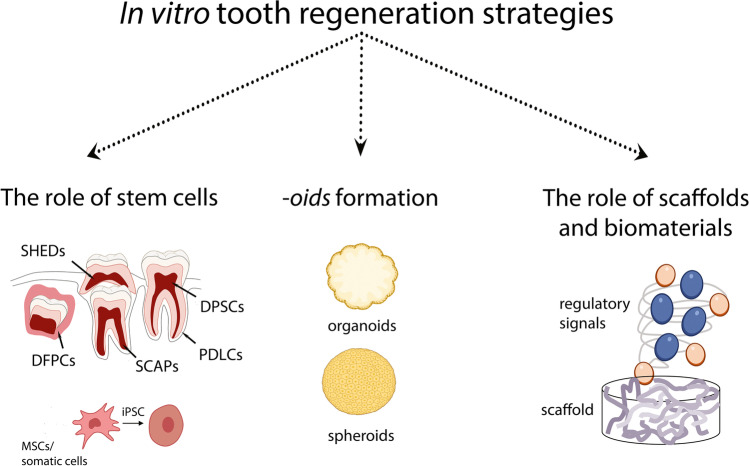

## Introduction

Regenerative medicine is a branch of medicine that focuses on repairing, replacing, or regenerating injured, diseased, or dysfunctional tissues. Current technologies in regenerative medicine rely extensively on advances in stem cell biology.

Stem cells (SCs) are undifferentiated cells capable of self-renewal and differentiation into more specialized cells. Based on their differentiation potential, SCs can be classified as totipotent, pluripotent, or multipotent [1]. Totipotent SCs can differentiate into both embryonic and extraembryonic tissues [2]. Pluripotent SCs (PSCs) can differentiate into the three embryonic germ layers – endoderm, mesoderm, and ectoderm [3]. Multipotent SCs, including the still debated mesenchymal SCs (MSCs), can differentiate into a limited number of specialized cells [4].

Because of their ability to self-renew and maintain their pluripotency given proper culture conditions, human PSCs have been a major focus of interest for studying tissue regeneration, modeling disease, and understanding tissue development [5]. PSCs called embryonic stem (ES) cells were initially derived from the inner cell mass of pre-implantation blastocysts [3, 6] and fetuses [7]. In the early years of this millennium, Kucia and collaborators identified, in mouse bone marrow [8] and human cord blood [9], a population of rare and very small cells positive for pluripotent markers and able to differentiate towards the three germ layers. They called them Very Small Embryonic-Like stem cells (VSELs). Since then, several groups have identified these cells in different adult tissues in both mice and humans [10], and although their existence is still debated and they are often called with different names (MAPCs, MUSE, and MIAMI cells) [11, 12], they represent an intriguing source of cells endowed with pluripotent features in the field of stem cell biology.

Another type of PSCs, induced pluripotent stem (iPS) cells, can be generated by reprogramming somatic cells by expressing four transcription factors – *Oct3/4*, *Sox2*, *Myc*, and *Klf4* [13, 14].

However, the use of PSCs in clinical applications has been challenged by ethical concerns, potential immunogenicity and tumorigenicity, and epigenomic instability [15–17]. Thus, during the past couple of decades, MSCs have become promising candidates for regenerative medicine and tissue engineering applications.

MSCs can be easily harvested, potentially from autologous grafts, and are ethically uncontroversial while also maintaining the ability to differentiate into osteocytes (bone), chondrocytes (cartilage), and adipocytes (fat) contingent on their exposure to particular factors in their microenvironment [18]. MSCs regulate tissue homeostasis through their secretome and replenish the cellular components of their niche [19]. From their initial discovery in the bone marrow, MSCs have been successfully isolated from most adult tissues, opening new avenues of research and development of therapeutic technologies [20–22]. Some MSCs of particular interest in regenerative medicine are derived from various dental tissues. These dental MSC populations are heterogeneous: some share similar mesenchymal properties with bone marrow MSCs while others have a restricted differentiation potency [23, 24]. Dental MSCs are important for tooth homeostasis and repair (MSCs of the periodontal ligament), as well as for dentine repair (dental pulp MSCs). Dental MSCs have recently been used in several clinical trials, mainly for restoring the tooth pulp, bone regeneration, and periodontitis treatment [25].

Although in vitro culture and transplantation of dental SCs into animal models has helped us identify various dental SCs populations and their replicative and differentiation potential, we need better in vitro methods to replicate in situ processes of human organ formation. While murine tooth development has been studied extensively, little is known about the spatiotemporal cues of human odontogenesis. A better understanding of such processes would allow for the development of targeted regenerative therapies.

This review outlines the discoveries in the oral stem cell field, focusing on the latest advances in biological research, such as the formation of organoids and spheroids and their possible contribution to the advancement of translational medicine.

## Overview of Different Types of Dental Stem Cells

Since the initial identification of dental stem cells in the early 2000s, recent advances in cell and molecular-based dentistry have led to promising developments in dental therapies aiming at repairing, replacing, and regenerating dental tissues. Moreover, new methods have been developed to study human tooth organogenesis.

Primary teeth start to form in the developing embryo between 6 and 8 weeks of gestation and originate from the interaction between the oral ectodermal epithelium and neural crest-derived mesenchyme [26–28]. This epithelial-mesenchymal interaction also controls the final differentiation of odontoblasts and ameloblasts during tooth generation [29, 30]. During odontogenesis, dental mesenchymal stem cells derive from peripheral nerve-associated glia and produce pulp cells and odontoblasts. Evidence of this embryonic origin was demonstrated in the mouse embryo in an elegant study published in 2014. The authors traced peripheral glia with a multi-color confetti mouse reporter to show that glia-derived cells contribute to dental mesenchymal stem cells during tooth organogenesis [31].

**Fig. 1:**
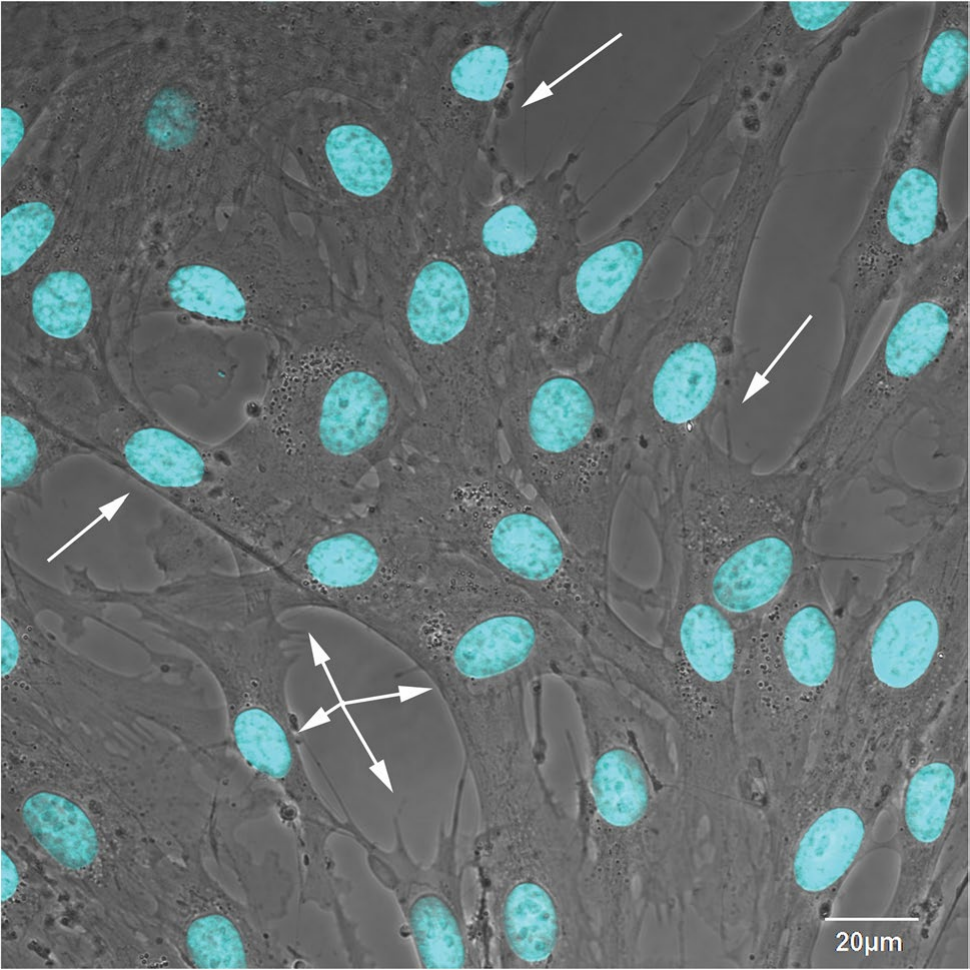
Phase contrast image of human dental pulp mesenchymal stem cells obtained from periosteum disaggregation as described in [21]. Nuclei are stained with DAPI (blue), arrows point to some of the mesenchymal DPSCs. Magnification 60X, bar: 20 μm

**Fig. 2:**
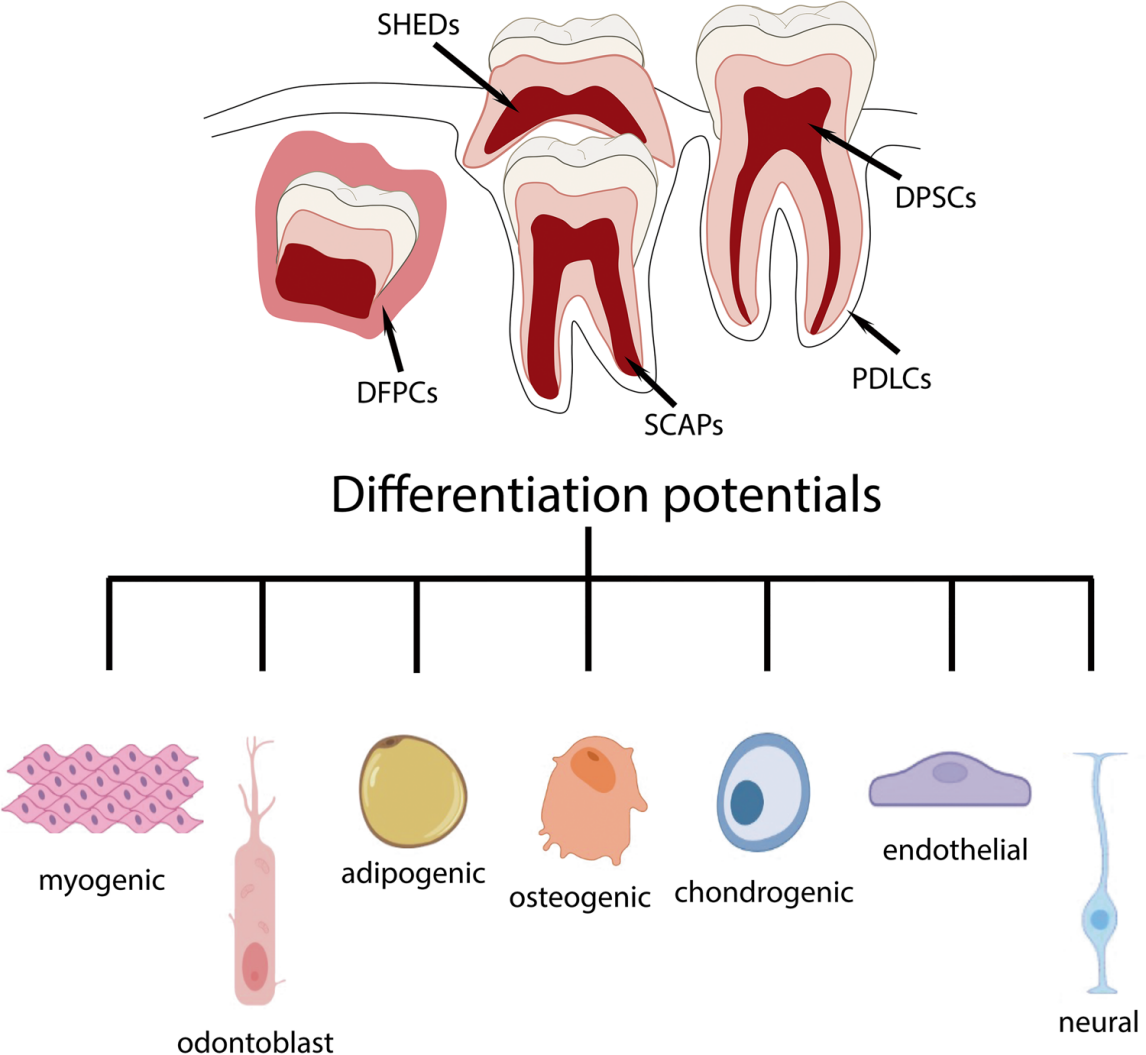
Differentiation potentials of dental stem cells (created with BioRender.com)

Teeth are one of the most accessible and least invasive sources of stem cells, and five subpopulations of dental and oral SCs have been identified (Table 1): dental pulp SCs (DPSCs) [32, 33] (Fig. 1), SCs from human exfoliated deciduous teeth (SHEDs) [34], periodontal ligament SCs (PDLSCs) [35], dental follicle progenitor SCs (DFPCs) [36], and SCs from apical papilla (SCAPs) [37, 38]. Although distinct, these populations have typical characteristics of MSCs: self-renewal capabilities and the ability to differentiate into at least three different lineages (Fig. 2) [39].

**Table 1:** Dental stem cells positivity to mesenchymal, ESCs, neural markers and differentiation potentials. Adapted from [40]

Stem cell type	Mesenchymal stem cell markers	ESCs markers	Neural markers	Differentiation potential
DPSC	CD29, CD34, CD44, CD59, CD105, CD73, CD90, CD105, CD117, CD146, CD166, CD271, STRO-1, CD271, SOX-10,	NANOG, OCT4, SOX-2, SSEA-3, SSEA-4	NESTIN, VIMENTIN, SOX-2	Osteogenic, chondrogenic, adipogenic, myogenic, neural, β-pancreatic, endothelial
SHED	CD44, CD105, CD73, CD90, CD146, STRO-1,	NANOG, OCT4, SSEA-3, SSEA-4,	NESTIN	Osteogenic, chondrogenic, adipogenic, odontogenic, neural, myogenic, hepatocytes
PDLSC	CD271, CD44, CD105, CD73, CD90, STRO-1	NANOG, OCT4, KLF4, SOX-2,	SLUG, NESTIN, NG2	Osteogenic, chondrogenic, adipogenic, neural, hepatocytes, β-pancreatic
DFPC	CD29, CD44, CD105, NOTCH-1		NESTIN, βIII TUBULIN, GFAP	Odontogenic, osteogenic, adipogenic, neural
SCAP	NOTCH-3, CD105, CD73, CD90, STRO-1, CD146, CD24, SURVIVIN	NANOG, OCT4,	NESTIN, GFAP	Odontogenic, osteogenic, chondrogenic, adipogenic, neural, hepatocytes

### Dental Pulp Stem Cells (DPSCs) and Stem Cells from Human Exfoliated Deciduous Teeth (SHED)

DPSCs are isolated from the dental pulp, extracted from teeth removed during routine dental procedures. DPSCs have a high proliferative capacity, differentiate into odontoblasts, osteoblasts, adipocytes, and chondrocytes in vitro, and can generate dentin and pulp-like tissues upon transplantation into immunodeficient mice [40–45]. When isolated from the dental pulp of exfoliated deciduous teeth, these cells are referred to as SHEDs. Similar to DPSCs, SHEDs can differentiate into many lineages in vitro and generate dentin and pulp-like tissues upon transplantation in mice. However, they show a significantly higher proliferation rate than DPSCs [34, 39, 45]. Of the various dental stem cell populations, both stem cell populations isolated from dental pulp - DPSCs and SHEDs - have been the primary source of cells used in periodontal clinical trials to regenerate dental pulp as well as bone and periodontal tissue [25, 46–48].

Moreover, due to their origin from the neural crest and their mesenchymal phenotype, DPSCs have been extensively studied for their potential uses beyond dentistry [49]. For example, DPSCs isolated from deciduous teeth can be differentiated in vitro into mature neurons or glial cells to replace dead neurons or injured peripheral nerves, form myelin, or provide support and protection for nerve cells after transplantation into the central nervous system [50, 51]. Evidence in studies using rats as animal models showed that DPSC grafts induce the survival of damaged motor neurons after spinal cord injury [52]. Other studies showed that the neurotrophic factors secreted by these cells influence the survival of dopaminergic neurons in both Alzheimer’s and Parkinson’s models [52, 53]. DPSCs have also been used as a therapeutic to repair myocardial infarction in nude rats where they reduced infarct size likely due to the secretion of proangiogenic and antiapoptotic factors [54]. Several in vitro studies have also described their ability to differentiate into hepatocyte-like cells [55] able to treat liver disease [56, 57], to differentiate into islet-like cell aggregates to treat diabetes [58], and to differentiate into cornea epithelium and stromal cells [59].

### Periodontal Ligament Stem Cells (PDLCSs)

PDLSCs are isolated from the perivascular wall of the periodontal ligament, which can be harvested from the roots of extracted teeth. PDLSC transplants in immunocompromised mice formed cementum/periodontal ligament (PDL)-like structures that supported periodontal tissue repair [60–62]. Unlike DPSCs, PDLSCs have only been used in clinical trials to regenerate their corresponding tissue [25].

### Dental Follicle Progenitor Stem Cells (DFPCs) and Apical Papilla Stem Cells (SCAPs)

Dental follicle progenitor SCs are isolated from dental follicle tissue surrounding the developing tooth. In vitro, DFPCs have osteogenic potential and can gain cementoblast features [36, 63]. Upon transplantation in rats, DFPCs produced a cement matrix with embedded cementoblast/osteocyte cells [64].

SCAPs are SCs derived from the apical papilla of immature permanent teeth. SCAPs have osteogenic/odontogenic differentiation potential in vitro, similar to DPSCs, but proliferate at a significantly higher rate [65]. Upon transplantation in immunodeficient mice, seeded SCAPs formed a vascularized dental pulp-like tissue in the root canal space and differentiated into osteoblast-like cells capable of producing dentin-like tissue [66].

### Tooth Regeneration Strategies

#### The Role of Stem Cells

Replacement of a missing tooth is a routine procedure requiring the placement of a dental implant or bridge into the jawbone. If necessary, this procedure is preceded by a bone graft to repair or rebuild the patient’s jawbone. However, current implant-based strategies for tooth replacement fail to reproduce a natural root structure, potentially leading to the loss of supporting bone due to peri-implantitis (or inflammation of gum and bone around dental implants). To address implant failure, new strategies are being investigated to regenerate teeth in vitro using SCs, biomaterials, and specific 3D culture conditions able to mimic, as faithfully as possible, the niche in which dental stem cells usually reside.

Several approaches have been used and tested to regenerate teeth [67]; these approaches have focused on targeted regeneration of individual tooth components such as dental pulp and dentin [for reviews see 68–70; for functional studies see 70–73], cementum [for reviews see 74, 75; for functional studies see 76–78], periodontium [for reviews see 79, 80; for functional studies see 81–83], and enamel [for reviews see 84; for functional studies see 85–88]. However, combining these strategies does not guarantee the successful regeneration of a viable tooth. In the mouse, dental structures have been obtained upon transplantation of recombined embryonic oral epithelium and adult mesenchyme directly into the adult jawbone or under renal capsules [89] or by implanting embryonic rat molar cells into the maxilla of adult mice [90, 91]. Such studies suggest the possibility of using stem cells for the regeneration of an entire tooth.

These pioneering studies lead the way to developing strategies to obtain a tooth in vitro by mimicking the in vivo environment. For example, the interaction between mesenchymal and embryonic epithelial cells in vitro can induce the formation of a primordial tooth, which, upon transplantation into the adult oral cavity, can develop into a mature tooth [92]. Another study described a method to replace missing teeth using a patient’s autologous gum cells first isolated and expanded in vitro and then combined with murine embryonic tooth mesenchyme stem cells to induce tooth formation [24]. Signals released by the mesenchymal stem cells triggered the differentiation of epithelial cells into appropriate specialized epithelial derivatives to induce the formation of complete teeth. Tissue-engineered teeth, like natural ones, can also be produced by inducing an odontogenic response in non-dental stem cells such as embryonic stem cells, neural and bone marrow-derived cells through exposure to the appropriate oral epithelial signals in culture [89].

Another approach for tooth regeneration uses iPS technology. Otsu and collaborators were able to differentiate mouse iPS cells into neural crest-like cells and eventually into odontogenic mesenchymal cells [93]. Similarly, a few years later, they collected and reprogrammed a patient’s somatic cells towards the ectodermal epithelial and neural crest-derived mesenchymal lineage. The recombination of these cell types followed by subsequent transplantation into the mouth demonstrated the possibility of forming a tooth germ and functional final tooth [94]. Interestingly, Cai and colleagues showed that integration-free human urine-induced pluripotent stem cells could regenerate patient-specific dental tissues and teeth thanks to a two-steps methodology in which cells were first differentiated towards the epithelial phenotype and then re-combined with embryonic dental mesenchyme. The authors could get tooth-like structures after only three weeks in culture [95]. As obtaining and maintaining dental stem epithelial cells can be difficult, Kim and collaborators recently overcame this problem by differentiating human ES and human iPS cells into epithelial-like stem cells, thanks to direct interaction with Hertwig’s epithelial root sheath/epithelial rests of Malassez cell line [96].

Although many parameters must be considered to regenerate a functional tooth, the methodology that involves the use of iPS cells seems to be the most promising one. These cells have optimal proliferation capacity and more potential in autologous transplantation than other cell types. If combined with biomaterials and different kinds of scaffolds, iPSCs could induce and support better dental development. Furthermore, the use of transwell membrane co-culture of dental epithelial and mesenchyme cells has proved to be a valuable methodology to effectively differentiate iPSCs in dental cells, as shown in 2019 [97].

### The Role of Scaffolds and Biomaterials

Tissue engineering, a discipline that combines principles of engineering and biology to restore and/or improve tissue function, has been an emerging and exciting field in dentistry. Tooth tissue engineering has the potential to overcome the limitations pertaining to two-dimensional (2D) SCs culture because it relies on the combination of three key elements – cells, scaffold, and the biological environment (regulatory signals) – for proper tissue regeneration. Initially, two tissue-engineering methodologies were used to regenerate a tooth. The first one involved the dissociation of tooth germ that can be seeded onto a tooth-shaped scaffold supporting tooth formation and is then transplanted to generate multiple complex tooth-like structures. The second approach relied on the interaction between epithelial and mesenchymal SCs obtained from primordial tooth germs or other sources, which induced tooth growth in culture [98].

Scaffolds and biomaterials are important factors supporting tissue regeneration. They must mimic, in vitro, the physiological environment necessary for cellular growth, expansion, and differentiation [99, 100]. Scaffolds must be biocompatible, ensure adequate diffusion of nutrients, and have porous structures that allow cell penetration. They should also prevent the production of non-toxic molecules and chronic inflammatory responses while delivering necessary regulatory signals in a controlled way to promote healing. Finally, scaffolds should be biodegradable, allowing their replacement by regenerated tissues [98, 101].

Over the years, many biodegradable and biocompatible biomaterials have been optimized to support the regenerative process. Biomaterials can be natural or synthetic. Natural biomaterials – such as natural polymers (collagen, laminin, elastin, chitosan, silk, platelet-rich plasma, bone sialoprotein, to name a few) – usually have low toxicity, are eco-friendly, and are cheaper than synthetic biomaterials. They are preferred for cell adhesion, cell-responsive degradation, proper cell signaling, and rapid degradation without immune rejection [98–101]. Tooth tissue engineering also uses synthetic materials – such as hydroxyapatite, polylactic acid, polyglycolic acid, polycaprolactone, and poly lactic-co-glycolic acid – which are often more flexible and elastic than natural ones. However, synthetic biomaterials are often not as conducive to recellularization and remodeling as natural ones. Thus, composite materials such as gelatin-chondroitin-hyaluronan tri-copolymer and polycaprolactone-gelatin-hydroxyapatite tri-copolymer are now preferred [98, 101].

The correct “niche” reconstitution to allow cells to proliferate and differentiate within a given scaffold implies integrating both the cells and the scaffold with suitable growth factors. These should favor, promote, and support tooth morphogenesis. The members of the transforming, epidermal, fibroblast, and insulin growth factor families, along with bone morphogenetic and WNT proteins, are the most widely used because they promote cell migration, proliferation, and differentiation. These regulatory signals support the development of the tissue-engineering tooth beyond odontogenesis [98].

Thanks to the continued development of new biomaterials for scaffolding and the increasing knowledge of the signals defining the dental niche, tissue engineering has made great strides in dentistry. For example, it was recently shown that a thermosensitive injectable hydrogel containing graphene oxide and chitosan represents an ideal scaffold for the growth of DPSCs by inducing the expression of factors specific for osteogenic differentiation, like Runt-related transcription factor 2 and osteocalcin [102]. Another research showed how DPSCs involved in dentin regeneration increase their odontogenic potential when cultured in chitosan scaffolds enriched with calcium-aluminate and 1α,25-dihydroxy vitamin D3 and how the composition, porosity, and organization in interconnected pore networks better supported this process [103]. The osteogenic potential of DPSCs was also evaluated in a porous composite scaffold based on chitosan-gelatin and nanohydroxyapatite enriched with fibrin glue and platelet-rich plasma. This particular “reconstructed niche” allowed the proliferation and differentiation of stem cells and induced the increased expression of markers specific for bone formation during the first weeks in culture [104]. Human platelet lysate was also used to evaluate the proliferation capacity of both SCAPs and PDLSCs on a synthetic scaffold fabricated from poly ‘lactic-co-glycolic’ acid. Human platelet lysate, whose effects are comparable to those obtained from the use of fetal bovine serum, represents a valid alternative for inducing dental stem cells to differentiate towards osteogenic lineages [105]. The dentin-pulp-enamel tissue complexes of the human tooth were mimicked by injecting different stem cells (i.e., human bone marrow stem cells, fat cells, and gingival epithelial cells) into a scaffold made of hydroxyapatite. This study demonstrates how these conditions can support and induce the early stages of tooth development [106].

Biomaterials are of enormous help in inducing cell growth and differentiation, as the studies cited above show. However, a relatively new field, bioprinting, is emerging to address the need to reconstruct complex 3D structures. Specific biomaterials such as those mentioned above and living cells become the ink needed to print scaffolds and structures on which cells are seeded. This technology is also applied to dentistry and a recent study demonstrated that DPSCs grown on a scaffold obtained with different biomaterials and printed with the 3D bioprinter show high cell viability and osteogenic differentiation as well as mineralization comparable to more canonical cell culture [107–109].

These advancements will play an essential role in creating a supportive microenvironment to preserve tissue function upon implantation properly. In addition, such advances in tooth tissue engineering offer a promising future for dental care and a better understanding of the biology of tooth formation.

### Spheroids and Organoids: New Perspectives in Dentistry

In the last decade, stem cell biology has made tremendous progress in the study and development of –oids (gastruloids, spheroids, organoids) from the in vitro three-dimensional (3D) culture of SCs to mimic the physiological properties and tissue architecture of embryonic stages, tissues and organs [110, 111].

Gastruloids obtained from ESCs offer a sophisticated model to study animal and human embryological development and diseases. Spheroids, developed in the 1970s [112] to study the effect of radiotherapy on tumor cells, are also used to induce embryoid bodies formation from PSCs and culture MSCs in 3D. While organoids, formed by self-organizing stem cells, differentiate towards lineages specific to the tissue/organ of interest [113]. They can be defined as 3D multicellular structures grown in vitro but able to mimic some of the complexity of the corresponding organ in vivo. Organoids can be grown from two types of cells, PSCs (ES and iPS) and SCs from adult organs. Their formation requires the use of biomaterials (i.e., hydrogels, Matrigel) and specific growth factors to direct cell differentiation towards the cell types that constitute the mimicked organ [114]. This allows studying both normal and pathological conditions, testing treatments, and evaluating the action of drugs and/or toxic compounds [114].

The interesting and surprising use of -oids technology occurs in dentistry. In 2007, scientists were able to form a tooth germ organoid using a combination of dental epithelium and mesenchyme cells obtained from mandibular tooth germs at the cap stage of mouse post-implanted embryos. The use of a biomaterial (i.e., collagen) allowed the authors to obtain a tooth germ-like structure capable of forming a bioengineered tooth upon transplantation [115].

Several groups obtained dental spheroids a few years ago using dental epithelial cells and MSCs in mouse and human models [116, 117]. In 2011, Berahim and colleagues grew spheroids from human periodontal ligament fibroblasts and then transplanted them onto membranes enriched with collagen and polyglycolic acid. They were able to demonstrate the ability of these cells to grow, proliferate and migrate in 3D [116]. A few years later, Natsiou and collaborators developed a technique to form the so-called “dentospheres” using dental epithelial SCs from the cervical loop area of the mouse incisor [118]. The 3D culture of the cells using two different media enriched by growth factors allowed the formation of spheroids of varying morphology and size. Their results defined the stemness and plasticity of dental SCs and their ability to be manipulated in these conditions.

Spheroids represent an intriguing tool to determine optimal culture conditions and biomaterials to induce 3D organization of dental SCs. In 2021, RAMAN spectroscopy was used to find the factors responsible for stem cell differentiation in spheroids formed by aggregation of human DPSCs. This research showed that the differentiation and acquisition of the 3D structure is mainly related to the diffusion of nutrients, morphogens, and growth factors within the culture medium [119].

Desirable culture conditions may also be achieved by co-culture of different types of tissues and/or cells. In 2020, Sano and collaborators co-cultured spheroids of human PDLCSs with vascular endothelial cells to induce periodontal tissue regeneration. They showed that the treatment with co-cultured spheroids led to new cementum formation after one or two months after transplantation. Also, the expression of stemness, vascular endothelial growth factors, and osteogenesis markers increased compared to the same cells grown in monolayer [120]. In 2017, Ono and collaborators obtained a bioengineered tooth in the canine model, physiologically similar to a normal tooth. The authors demonstrated that combining epithelial tissue with mesenchymal tissue or mesenchyme cells, or epithelial cells and mesenchyme tissue yields better tooth formation than combining epithelial and mesenchyme cells. The combination and culture of these cells and tissues formed a tooth germ organoid that, transplanted in the canine mandible, developed into a bioengineered tooth characterized by enamel, dentin, and pulp tissue several weeks later [121].

A bio-engineered tooth was also obtained in *Sus scrofa*. Pigs are helpful animal models for studying human diseases, xenotransplantation, and tooth formation due to the similarities shared with humans. Wang and collaborators combined isolated epithelial and mesenchymal cells that formed a tooth organoid after transplantation in mouse sub renal capsules and jawbone, which later developed into a large-size tooth [122].

Jeong and colleagues recently developed dentin-pulp-like organoids by cultivating human DPSCs with Matrigel using appropriate differentiation media. After harvesting and characterization, these organoids were dissociated and successfully reorganized into more dentin pulp-like organoids. These revealed characteristics of both SCs and differentiated odontoblast-like cells, thus representing a good starting point for future use of these structures in human dentistry [123].

Organoids have been established for the salivary gland, lingual epithelium, and taste buds and show similar characteristics to those of the corresponding organs [124]. Salivary gland organoids can be obtained with two methodologies. The first induces PSCs to differentiate towards oral ectoderm in a 3D culture enriched with several growth factors and cytokines to promote the salivary gland morphogenesis [125]. The second requires the incubation of salivary gland progenitors in 3D scaffolds to promote the formation of the gland structure thanks to several growth factors [126, 127]. Salivary organoids have also been obtained thanks to magnetic 3D bioprinting using DPSCs and neural crest-derived mesenchymal stem cells as “ink” [128]. This technique involves using magnetic nanoparticles tagged to the cells to print 3D spheroids able to induce the formation of salivary gland epithelium. Transplantation into ex vivo models was useful to study salivary gland morphogenesis and growth.

Lingual organoids have been obtained by cultivating lingual stem cells in Matrigel with specific cytokines and growth factors. This setup generated rugged round-shaped organoids with a reticulated cell arrangement and round-shaped organoids with concentric cell arrangements [129, 130]. The latter had a morphology typical of filiform papillae found in the tongue. The authors of these studies were able to transplant these organoids into recipient mouse tongues and follow their maturation in both normal and pathological conditions.

Taste bud organoids can be obtained from taste bud stem cells or circumvallate papillae in a 3D culture enriched with several factors able to support and induce their formation, maintaining phenotypic characteristics similar to native tissue [131].

Overall, dental spheroids and organoids present an excellent opportunity for the advancement of oral biology research as well as dental practices. These 3D cultures provide an accessible system for modeling human organogenesis, modeling diseases, and regenerative medicine. They allow complex interactions between cells, the flow of signaling molecules and nutrients, and self-organization (specific to organoids) to help scientists model and understand dental physiology. Spheroids and organoids can emulate in vivo conditions more closely than 2D cultures [116, 132, 133] and provide a better and more accessible understanding of human odontogenesis than animal models. 3D cultures support cell-cell and cell-microenvironment interactions that play a fundamental role in regulating cell proliferation, migration, and differentiation. They also promote the development and physiology of a particular pathology, which presents some limitations if studied with the traditional 2D culture. An exciting discipline applied to -oids is mechanobiology, which studies the roles of mechanical forces during cell migration, cell differentiation, cell adhesion to substrates and, for example, extravasation of cancer cells [134]. Some known mechanotransducers are the two transcriptional regulators, YAP and TAZ, which sense mechanical cure within cells (and during cell-environment interactions) and respond to them in a cell-specific manner [135]. For example, YAP and TAZ induce transient stem cell proliferation when intestinal organoids grow on a hydrogel scaffold with defined stiffness and composition. If the organoid increases in size, YAP and TAZ are disabled, cells lose their stemness, and the organoid undergoes apoptosis and necrosis. However, when the hydrogel composition is more plastic, stem cells are preserved, and the activity of YAP/TAZ is localized in a specific area of the organoid resembling the intestinal crypts [136, 137]. Very recently, it was demonstrated that the use of a scaffold composed of hyaluronan induced the differentiation of human DPSCs towards the osteogenic lineage thanks to the activation of the YAP/TAZ pathway [138].

The 3D cell spatial organization studies could also help assemble patient-specific organoids composed of patients’ cells to develop and study dental anomalies or diseases.

For example, organoids have been used to study oral tumors [139] to capture and maintain the original tumor’s composition. Patient-derived organoids for disease modeling also provide an opportunity for personalized medicine. Finally, organoids can be derived from small amounts of a patient’s cells, which would then expand and differentiate in vitro, thus providing autologous sources of dental stem cells for transplantation and regeneration of damaged tissues. Although oral spheroid and organoid technologies have developed significantly in the past decade, these 3D structures still lack many features necessary for organ function [140]. Therefore, it is unknown how faithful they are in representing in vivo dental structures or whether they could replace original oral components upon transplantation.

Despite needing further improvements, organoid technologies represent an important prospect for personalized medicine based on patients’ specific needs.

## Discussion

Organoids represent one of the most futuristic cellular models for biomedical research. They can reproduce the architecture and complexity of various organs and tissues. Also, they can be implemented for multiple uses, such as in vitro testing of therapies before administration, modeling oncological and rare genetic diseases, regenerative medicine, and transplantation procedures.

Many pioneering studies lead the way in forming and using these 3D structures. For example, at the beginning of this millennium, developmental biologist Yoshiki Sasai and his team proposed that ES cells might be able to self-organize into 3D structures that resemble small functional organs in response to appropriate culture conditions. They obtained a 3D little brain, optic cup, neocortex, cerebellum, hippocampus, adenohypophysis, ventral telencephalon, and pituitary gland [141]. These results have paved the way for further advances in this field, as demonstrated by recent research on recording oscillatory waves from brain organoids [142].

Human organoids were obtained during the following years for intestine, kidney, pancreas, liver, brain, and retina [143], opening innovative approaches for developing new drugs, toxicological assays, and therapeutic opportunities. The possibility of using organoids to study neoplastic transformation and tumor formation in a petri dish [144] and using patient cells to obtain patient-specific organoids to recreate diseases in vitro are undoubtedly significant [145]. For example, the production of patient-specific intestinal organoids obtained from culturing cells on specific biomaterials allowed scientists to develop drugs to treat cystic fibrosis, thus improving patients’ symptoms and quality of life [146, 147]. Organoid technology is also employed to treat deafness, an increasingly widespread pathology that affects all demographics. The 3D cultures of SCs from the cochlea allowed the identification of molecules capable of stimulating the expansion of endogenous SCs [148], which can then be induced to differentiate into sensory hair cells [149]. A similar approach involving the use of hydrogel and in silico selection of new molecules capable of stimulating endogenous SCs led to another success: the identification of prostaglandin E2 as a natural modulator of the inflammatory process capable of stimulating the expansion of skeletal muscle SCs in vitro and muscle regeneration in vivo [150]. This means that there is a population of quiescent SCs that decreases with age in skeletal muscles. Their stimulation resumes expansion leading to rejuvenation of muscle function. This is of particular importance for counteracting the muscular weaknesses associated with old age and many other morbidities.

One of the most recent and undoubtedly interesting advances regards how organoids technology can be used to study SARS-CoV-2 infections in human cells. This can model how the virus infects various cell types and can aid in developing a vaccine capable of stopping the virus’s progression [151].

Due to their potential, the human cell atlas scientific community is working on an atlas of organoids to provide the necessary tools to improve current protocols and validate existing ones [152]. Sophisticated techniques such as spatial profiling and single-cell sequencing will be used to achieve this goal. Spatial profiling will allow the characterization of the organoids in their 3D structure. At the same time, single-cell sequencing will define both the cellular composition and the genes expressed by each organoid cell. Intestine and brain organoids will be the first to be implemented because a fair number of protocols are already available although the goal is to extend the project to all other organs as well. Organoids can become crucial for the study of pathology and regeneration of oral and maxillofacial tissue and precision therapies targeting oral cancers. Moreover, hopes for the future lie in the possibility of their use to investigate the etiology of rare and/or heritable diseases whose origin is often associated with dental anomalies. An example is the congenital ectodermal dysplasia characterized by anomalies in ectodermal derivatives such as teeth, nails, sweat and salivary glands, hair, cranio-facial structure, and other body parts [153].

## Hype and Hopes of Organoids/Spheroids Research

As already stressed, studying these 3D structures opens many perspectives for the translational medicine of the future. Although the mechanism used by stem cells to reproduce in vitro what they would typically do in vivo is now quite clear, the way stem cells self-assemble into organoids/spheroids is still under investigation. This aspect still represents a weak point of this technology. Until the mechanisms of these systems can be completely controlled, there will be a limitation in regard to experimental reproducibility, which is an essential condition for their use, for example, in developing new drugs, toxicological and therapeutic assays. Thus, it will be necessary to improve the culture techniques by choosing suitable biomaterials and scaffolds that guarantee the symmetrical and three-dimensional structure maintenance, the modulation of adequate morphogens, and the delivery of nutrients and growth factors. Basic biology research is proceeding very fast in this field and, beyond promises that can create false expectations, in a few years, we will be able to have complete control even of this fascinating aspect of biology: the assembly of cells in structures which, thanks to the cell-cell, cell-environment, cell-substances interactions that promote and maintain growth, allow us to recapitulate the development of an organ or embryonic development.

Faced with the considerable potential of spheroid/organoid research, it is necessary to consider the ethical implication of this technology, as it often requires the use of ES and fetal cells for research. It will also be necessary to discuss the ethics of gaining informed consent to create patient-specific organoids and their preservation [110, 154]. The latest innovations promise to revolutionize regenerative medicine by overcoming the current biological obstacles (the long timeline to get validated therapies is not always due to bureaucratic problems) and developing several therapies. To date, most of the SC-based therapies have not yet obtained approval, and there are hundreds of clinical trials registered to await marketing approval by the US Food and Drug Administration. The organoids technology, along with gene editing and genetic reprogramming, promises to revolutionize biomedical research in the upcoming years. In addition, it will significantly improve therapies for clinical conditions with unmet clinical needs.
